# Association Between VEGF Expression and Diffusion Weighted Imaging in Several Tumors—A Systematic Review and Meta-Analysis

**DOI:** 10.3390/diagnostics9040126

**Published:** 2019-09-23

**Authors:** Hans-Jonas Meyer, Andreas Wienke, Alexey Surov

**Affiliations:** 1 Department of Diagnostic and Interventional Radiology, University of Leipzig, 04103 Leipzig, Germany; alexey.surov@medizin.uni-leipzig.de; 2 Institute of Medical Epidemiology, Biostatistics, and Informatics, Martin-Luther-University Halle-Wittenberg, 06112 Halle (Saale), Germany; andreas.wienke@medizin.uni-halle.de

**Keywords:** meta-analysis, DWI, ADC, VEGF

## Abstract

To date, only a few studies have investigated relationships between Diffusion-weighted imaging (DWI) and Vascular endothelial growth factor (VEGF) expression in tumors. The reported results are contradictory. The aim of the present analysis was to review the published results and to perform a meta-analysis regarding associations between apparent diffusion coefficients (ADC) derived from DWI and VEGF expression. MEDLINE library was screened for relationships between ADC and VEGF expression up to January 2019. Overall, 14 studies with 578 patients were identified. In 10 studies (71.4%) 3 T scanners were used and in four studies (28.6%) 1.5 T scanners. Furthermore, seven studies (50%) had a prospective design and seven studies (50%) had a retrospective design. Most frequently, prostate cancer, followed by rectal cancer, cervical cancer and esophageal cancer were identified. The pooled correlation coefficient of all tumors was *r* = −0.02 [95% CI −0.26–0.21]. ADC values derived from routinely acquired DWI do not correlate with VEGF expression in various tumors. Therefore, DWI is not sensitive enough to reflect angiogenesis-related microstructure of tumors.

## 1. Introduction

Diffusion-weighted imaging (DWI), quantified by apparent diffusion coefficients (ADC) besides diagnostic potential can also provide information regarding tumor microstructure [1,2,3,4]. This method utilizes the constant random movement of water molecules, called Brownian motion [4]. ADC is widely acknowledged to be mainly influenced by the cellularity of tumors and is inversely correlated with cell density in tissues [3]. The principle of this is that the cell membrane might hinder the water movement and, therefore, lead to a restriction of diffusion [5]. However, important factors are not only cell count but also cell size, cell nucleus size, and membrane permeability [5]. Moreover, it was shown that water molecules are also hindered by extracellular components, such as collagen fibers and extracellular matrix [6].

DWI is usually acquired by two b-values, a low one, usually 0 s/mm² and a high one, usually 800–1000 s/mm² [1,7]. The low signal intensity of DWI, up to 200 s/mm² is more sensitive to perfusion than the latter [1,7]. There is an ongoing debate on whether ADC values can also reflect perfusion related tumor features, such as vessel density [8,9,10]. Presumably, more water molecules can move freely and particularly fast within vessels. Moreover, it was hypothesized that ADC is even has the capacity to reflect factors influencing vascular angiogenesis, for example, expression of vascular endothelial growth factor (VEGF) [11,12].

Tumor angiogenesis is a hallmark, which provides oxygen and nutrients to tumor cells during cancer progression and metastasis [13]. VEGF has been generally regarded as a key factor in angiogenesis [14]. It is a protein family consisting of five subtypes with the regulation of the vessel cells by three cell membrane receptors [14].

The inhibition of VEGF-A with bevacizumab was the first angiogenesis-related tumor treatment, which nowadays is used for several different tumor entities [14,15]. Functional imaging biomarker guidance of VEGF treatment might be crucial due to the fact that anti-VEGF therapy might not primarily lead to shrinkage of the tumor, which could be assessed by morphological imaging, but to a devascularization of the tumor assessable only by functional imaging.

The associations between ADC and VEGF have been elucidated in preliminary small studies with incoherent results. Presumably, if routinely acquired ADC values are correlated with VEGF expression in tumors, this might also establish the opportunity for DWI to display treatment response to anti-angiogenesis therapy, which was previously shown in xenograft studies [11,16].

Therefore, the purpose of the present systematic review and meta-analysis was to review the published studies and to provide data of possible associations between ADC and VEGF expression in several tumors.

## 2. Materials and Methods

### Data Acquisition

MEDLINE and SCOPUS library were screened for associations between ADC values and VEGF expression up to September 2019. The following search words were used: ADC OR apparent diffusion coefficient OR DWI OR Diffusion weighted imaging AND VEGF OR vascular endothelial growth factor. Overall, 68 articles were identified throughout this search process.

The primary endpoint of the systematic review was the correlation between VEGF expression and ADC derived from DWI.

Studies (or subsets of studies) were included if they satisfied all of the following criteria: (1) patients with histopathologically confirmed tumors and expression analysis of VEGF on immunohistochemical stained specimens (2) DWI quantified by ADC (3) correlation analysis. 

Exclusion criteria were (1) systematic review (2) case report (3) treatment prediction or histopathology performed after treatment (4) non-English language (5) xenograft or mouse model studies.

**Figure 1:**
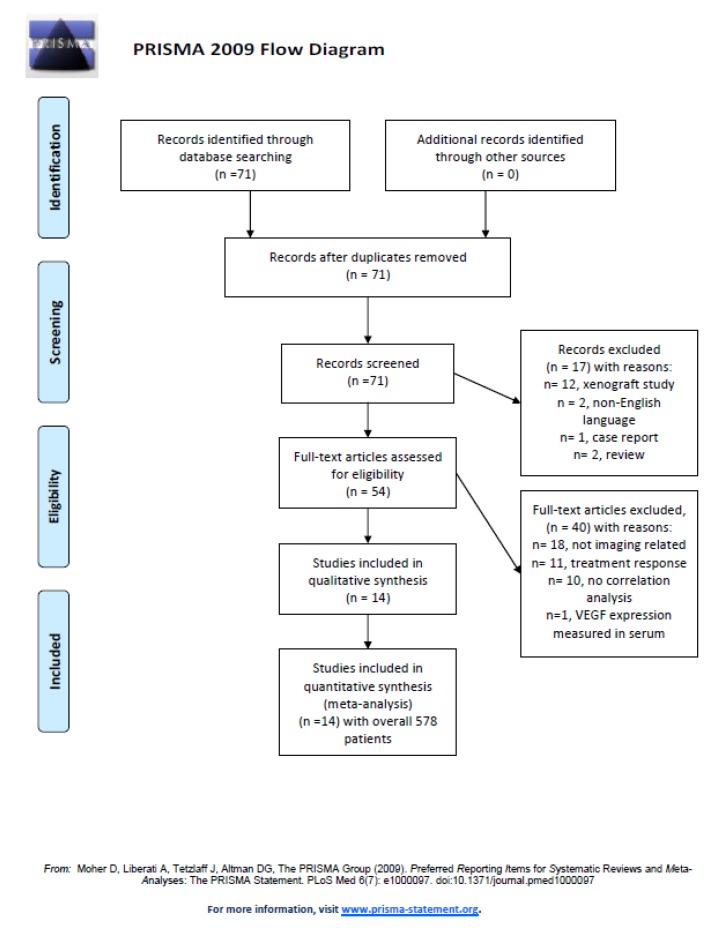
Preferred Reporting Items for Systematic Reviews and Meta-Analyses (PRISMA) flow chart. An overview of the paper acquisition. Finally, 14 articles were suitable for the analysis.

After thorough review 14 articles were suitable for the present meta-analysis [17,18,19,20,21,22,23,24,25,26,27,28,29,30]. The Preferred Reporting Items for Systematic Reviews and Meta-Analyses (PRISMA) statement was used for the research [31]. Figure 1 displays the PRISMA flow chart of the paper acquisition.

The following data were extracted from the literature: authors, year of publication, study design, tumor entity, number of patients, MRI scanner, b-values of DWI and correlation coefficients.

**Figure 2:**
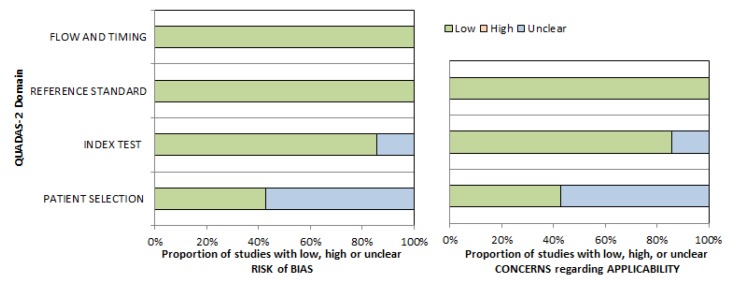
Quality Assessment of Diagnostic Studies (QUADAS-2) quality assessment of the included studies. Most studies showed an overall low risk of bias.

The methodological quality of the acquired studies was independently checked by two observers (HJM and AS) using the Quality Assessment of Diagnostic Studies (QUADAS 2) instrument, according to previous descriptions [32]. Figure 2 displays the QUADAS results. Most studies showed an overall low risk of bias.

Associations were analyzed by Spearman’s correlation coefficient. The Pearson’s correlation coefficients in some studies were converted into Spearman’s correlation coefficients, as reported previously [33].

Furthermore, the meta-analysis was undertaken by using RevMan 5.3 (Computer Program, version 5.3, The Cochrane Collaboration, 2014, The Nordic Cochrane Centre, Copenhagen, Denmark). Heterogeneity was calculated by means of the inconsistency index *I*^2^ [34,35]. Additionally, DerSimonian and Laird random-effects models with inverse-variance weights were used without any further correction [36].

## 3. Results

Overall, the collected 14 articles included 578 patients. In 10 studies (71.4%) 3 T scanners were used, and in four studies (28.6%) 1.5 T scanners. Furthermore, seven studies (50%) had a prospective design, seven studies (50%) had a retrospective design (Table 1). Table 2 summarizes the included tumor entities. Most frequently, prostate cancer, followed by rectal cancer, cervical cancer and esophageal cancer were identified.

**Figure 3:**
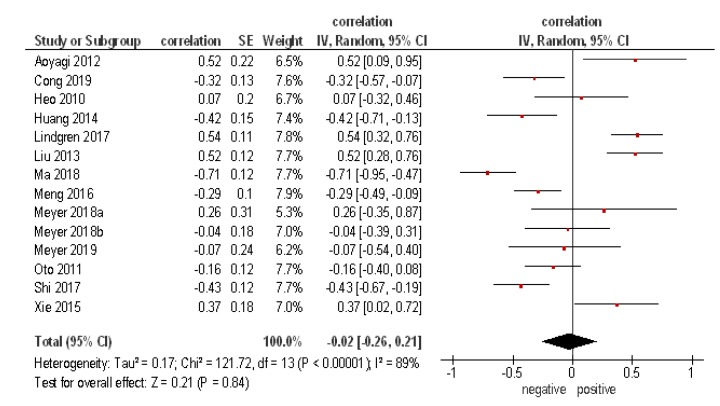
Forrest plots of the correlation coefficients between ADC values and VEGF expression. Overall, 14 studies comprising 578 patients. The pooled correlation coefficient was *r* = −0.02 [95% CI −0.26–0.21].

The pooled correlation coefficient between ADC and expression of VEGF *r* = −0.02 [95% CI −0.26–0.21], heterogeneity Tau² = 0.17, I² = 89 (Figure 3).

**Figure 4:**
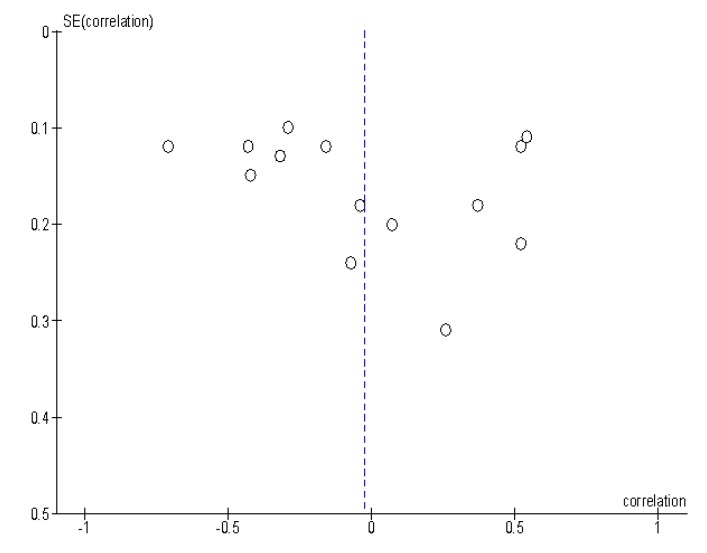
Funnel plot of the publication bias. There is no significant publication bias identified.

The Egger test does not support any linear association between correlation and its weighted standard error (*p* = 0.255) which speaks against publication bias (Q = 121.72), which is supported by the funnel plot (Figure 4).

## 4. Discussion

The present systematic review and meta-analysis did not find significant associations between ADC values and the expression of VEGF in tumors.

We identified a positive correlation for four studies including esophageal cancer, ovarian cancer, cervical cancer and pancreatic cancer. On the contrary, there were four studies with an inverse correlation including hepatocellular carcinoma, prostate cancer, rectal cancer and thyroid cancer. In five studies, there were no relationships between VEGF expression and ADC. These findings resulted in an overall non-existing association.

Previously, numerous investigations showed that ADC inversely correlated with cellularity in different malignant and benign lesions [3]. Moreover, it was widely acknowledged that DWI may discriminate tumor grades and tumor subtypes. For example, it was shown that benign lesions tended to have higher ADC values than malignant tumors [37,38].

Furthermore, according to the literature, ADC can also reflect other histopathological features, such as expression of proliferation factor Ki67, epidermal growth factor receptor expression and hypoxia-inducible factor 1-alpha [25,26,39].

However, as mentioned above, there are inconclusive results regarding possible associations between ADC and VEGF expression [17,22,24]. Hypothetically, a positive correlation between the parameters may exist. The rationale is that with higher VEGF expression there are more vessels and, thus, there are more fast protons within the vessels reflected by a higher ADC value.

VEGF is a key factor of tumor neoangiogenesis [14]. It has been shown that overexpression of VEGF is an overall indicator of poor survival in various tumor entities emphasizing its clinical importance [40,41,42]. Therefore, it may be beneficial, when imaging can correctly predict VEGF expression of tumors enabling a non-invasive and serial approach compared to bioptic samples. 

However, the direct association between VEGF expression and vascularity of tissues and, thus, the overall perfusion is complex. So far, there were no differences in colorectal cancers with high VEGF expression compared to tumors with low expression in regard to microvessel density [43]. Yet, in other studies, a moderate to strong correlation was identified between VEGF expression and microvessel density in several tumors [44,45,46]. In a recently published preliminary study investigating head and neck cancer, no correlation between microvessel density and ADC values was identified, which corroborates the present results that DWI is not able to reflect perfusion related histopathology features of tumors [47].

When ADC values would be sensitive enough for tissue alterations caused by VEGF expression, predominantly vessel growth and vessel density, DWI may aid in treatment response evaluation to VEGF targeted therapy. In fact, this has been shown in previous studies, for example, in glioblastoma patients and in a glioma experimental tumor model [48,49]. However, there were also reports indicating that DWI might not be sensitive in this regard [9]. Clearly, more studies are needed to validate these findings.

Intravoxel incoherent motion (IVIM), as an advanced DWI technique was introduced, which takes advantage of the perfusion related signal intensity [7]. By using several low b-values up to 200 mm²/s, IVIM can provide perfusion related parameters like perfusion fraction f and pseudo diffusion D*, which might be more sensitive to predict VEGF expression and vessel density of tumors [7]. However, the acquisition of IVIM and perfusion parameters is associated with several problems. IVIM protocols take a longer time duration of the sequence, which might hinder the translation into clinical routine. Furthermore, there is still lack of standardization of this technique. This fact hinders the external validations of the reported results.

Possible clinical implications of the present results are that ADC values derived from clinical routine DWI are not able to reflect VEGF expression in tumors. Further on, ADC values might, therefore, not be capable of predicting treatment response assessment with VEGF-targeted therapy.

There are several limitations of the present analysis to address. Firstly, it comprised half of the retrospective studies with known inherent potential bias. Secondly, there were not enough studies to perform tumor-specific sub analyses. Presumably, the heterogeneity shown in the analysis could be induced by differences in tumor types. Thirdly, there were different scanner types and DWI protocols, which have an influence on ADC values and consequently might result in possible bias. Fourthly, there might be possible publication bias, as it is known that negative studies are less likely to be published.

## 5. Conclusions

The present analysis showed that ADC does not correlate with the expression of VEGF and, therefore, cannot be used as a surrogate marker for this histopathological parameter in tumors using a routinely acquired DWI.

## Figures and Tables

**Table 1:** Overview of the included studies.

Author, Year	Country	Design	Number of Patients	Tumor Entity	Field Strength (T)	*b*-Values (s/mm²)
Aoyagi et al. 2012 [17]	Japan	prospective	17	Esophageal cancer	1.5	0;1000
Cong et al. 2019 [30]	China	retrospective	52	Esophageal cancer	3	0;800
Heo et al. 2010 [18]	South Korea	retrospective	27	Hepatocellular carcinoma	1.5	0;1000
Huang et al. 2014 [19]	China	retrospective	36	Hepatocellular carcinoma	3	0;800
Lindgren et al. 2017 [20]	Finland	prospective	40	Ovarian cancer	3	0;300;600
Liu et al. 2013 [21]	China	prospective	56	Cervical cancer	1.5	0;100;0;3000
Ma et al. 2018 [22]	China	prospective	39	Prostate cancer	3	0;800
Meng et al. 2016 [23]	China	prospective	91	Rectal cancer	3	0;800
Meyer et al. 2018 [24]	Germany	retrospective	11	Rectal cancer	3	0;1000
Meyer et al. 2018 [25]	Germany	retrospective	32	Head and neck cancer	3	0;800
Meyer et al. 2018 [26]	Germany	retrospective	18	Cervical cancer	3	0;1000
Oto et al. 2011 [27]	USA	retrospective	73	Prostate cancer	1.5	0;1500
Shi et al. 2017 [28]	China	prospective	58	Thyroid cancer	3	0;1000
Xie et al. 2015 [29]	China	prospective	28	Pancreatic cancer	3	0;1000

**Table 2:** Overview of the included tumor entities.

Tumor Type	*n* (%)
Prostate cancer	112 (19.4)
Rectal cancer	102 (17.7)
Cervical cancer	74 (12.8)
Esophageal cancer	69 (11.9)
Hepatocellular carcinoma	63 (10.9)
Thyroid cancer	58 (10.0)
Ovarian cancer	40 (6.9)
Head and neck cancer	32 (5.5)
Pancreatic cancer	28 (4.9)
Total	578 (100)

## Abbreviations

DWI	Diffusion-weighted imaging
ADC	Apparent diffusion coefficient
VEGF	Vascular endothelial growth factor
IVIM	Intravoxel incoherent motion
